# Geriatric Nutritional Risk Index Plays Important Role in Predicting In‐Hospital Mortality in Patients With Severe Fever With Thrombocytopenia Syndrome: A Multi‐Center Observational Study

**DOI:** 10.1002/jmv.70252

**Published:** 2025-02-18

**Authors:** Tingyu Zhang, Yanli Xu, Ziruo Ge, Di Tian, Chenxi Zhao, Qi Zhao, Ling Lin, Zhensheng Liu, Zhihai Chen

**Affiliations:** ^1^ National Key Laboratory of Intelligent Tracking and Forecasting for Infectious Diseases, Beijing Ditan Hospital Capital Medical University Beijing China; ^2^ Department of Infectious Diseases Yantai Qishan Hospital Yantai China; ^3^ Department of Cardiology, Cardiovascular Center, Beijing Friendship Hospital Capital Medical University Beijing China; ^4^ Department of Infectious Diseases Qing Dao No. 6 People's Hospital Qingdao China

**Keywords:** geriatric nutritional risk index, in‐hospital mortality, severe fever with thrombocytopenia syndrome

## Abstract

Geriatric nutritional risk index (GNRI) has been proposed as a reliable indicator of nutritional state and, when decreased, is closely associated with the severity and mortality risk of infectious disease. The current study retrospectively recruited patients who were admitted for SFTS from January 1, 2011 to January 1, 2024 at six medical centers. Two hundred and eighty‐two patients with SFTS who met the study protocol were finally enrolled in this study. Sixty patients suffered in‐hospital death during hospitalization, with a mortality rate of 21.3%. After adjustment of multiple models, GNRI remained a significant predictor of in‐hospital death, either examining HR by evaluating 1‐unit decrease of GNRI or by taking the higher median of GNRI as reference (all *p* < 0.05). GNRI displayed a moderate‐to‐high strength in predicting in‐hospital death, with an area under the receiver operating characteristic curve (AUC) of 0.791 [95% confidence interval (CI) 0.725–0.857, *p* < 0.001]. The addition of GNRI to a former established model exhibited significant improvement in the predictive value for in‐hospital death. GNRI, an important indicator simply calculated from ALB and BMI, is significantly and independently related to the risk of in‐hospital death in patients with SFTS.

**Abbreviations**
ALB: albumin
ALT: alanine aminotransferase
APTT: activated partial thromboplastin time
AST: aspartate aminotransferase
AUC: area under the ROC curve
BMI: body mass index
BUN: blood urea nitrogen
CI: 95% confidence interval
CK: creatine kinase
CRP: C‐reactive protein
DBIL: direct bilirubin
DBV: Dabie bandavirus
FBG: fasting blood glucose
GNRI: geriatric nutritional risk index
HGB: hemoglobin
HR: hazard ratio
IDI: integrated discrimination improvement
LDH: lactic dehydrogenase
LYM: lymphocyte count
LYM%: lymphocyte percentage
MONO: monocyte count
MONO%: monocyte percentage
NEU: neutrophil count
NEU%: neutrophil percentage
NRI: net reclassification improvement
PLT: platelet count
PT: prothrombin time
ROC: receiver operating characteristic
SFTS: severe fever with thrombocytopenia syndrome;
SFTSV: severe fever with thrombocytopenia syndrome virus
TBIL: total bilirubin
UA: uric acid
WBC: white blood cell count

## 1 Introduction

Severe fever with thrombocytopenia syndrome (SFTS) is a severe hemorrhagic infectious disease caused by the severe fever with thrombocytopenia syndrome virus (SFTSV), also named Dabie bandavirus (DBV) now, a single‐stranded negative‐sense RNA virus which was isolated by Yu et al. in 2011 and identified as a member of the Bandavirus genus within the Phenuiviridae family [1, 2]. DBV is primarily transmitted through tick bites, but human‐to‐human transmission has also been reported. Additionally, there have also been reports of animal‐to‐human transmission in recent years [3, 4, 5]. SFTS was first identified in China in 2009 and has since become prevalent in countries such as China, South Korea, Japan, and Vietnam [6, 7, 8, 9]. This disease commonly occurs in hilly, mountainous, and forested areas and primarily manifests as fever, leukopenia, thrombocytopenia, encephalopathy, and multiple organ damage. SFTS can lead to multiorgan failure in a short period, and the fatality rate is extremely high. In addition, there are currently no specific anti‐DBV drugs. Therefore, early identification of risk predictors of severe illness plays a crucial role in improving the prognosis of SFTS patients.

Malnutrition has been shown to be associated with poor prognosis in patients with various diseases, particularly among the elderly, where malnutrition is quite common. The geriatric nutritional risk index (GNRI) was first proposed by Bouillanne et al. and serves as a screening tool for nutrition‐related risk in elderly patients. It has been demonstrated that GNRI is closely associated with the severity of malnutrition and mortality [10, 11]. The GNRI is simply calculated using serum albumin (ALB) and body mass index (BMI) [12], making it a practical tool for assessing nutritional status, and has been validated in numerous studies involving rehabilitation and long‐term care settings. In recent years, GNRI has been used as an indicator of nutritional status to predict the severity and risk of death in various diseases. Certain research have indicated that GNRI is significantly related to clinical outcomes in patients with heart failure, stroke, end‐stage renal disease, malignancies, and other ailments [13, 14, 15, 16]. Recent studies have pointed out that GNRI can predict poor outcomes in elderly patients with COVID‐19 and serves as an independent risk factor for in‐hospital mortality [17]. Additionally, GNRI has been proven to be closely associated with poor outcomes in patients with infectious diseases such as sepsis and liver abscesses [18, 19].

However, studies targeting at the impact of nutritional status on the prognosis of elderly patients with SFTS are currently lacking. This study aims to explore the relationship between GNRI levels and clinical outcomes in elderly patients with SFTS, so as to offer useful information for early identifying high risk patients who are susceptible to adverse prognosis.

## 2 Methods

### 2.1 Study Population

**Figure 1:**
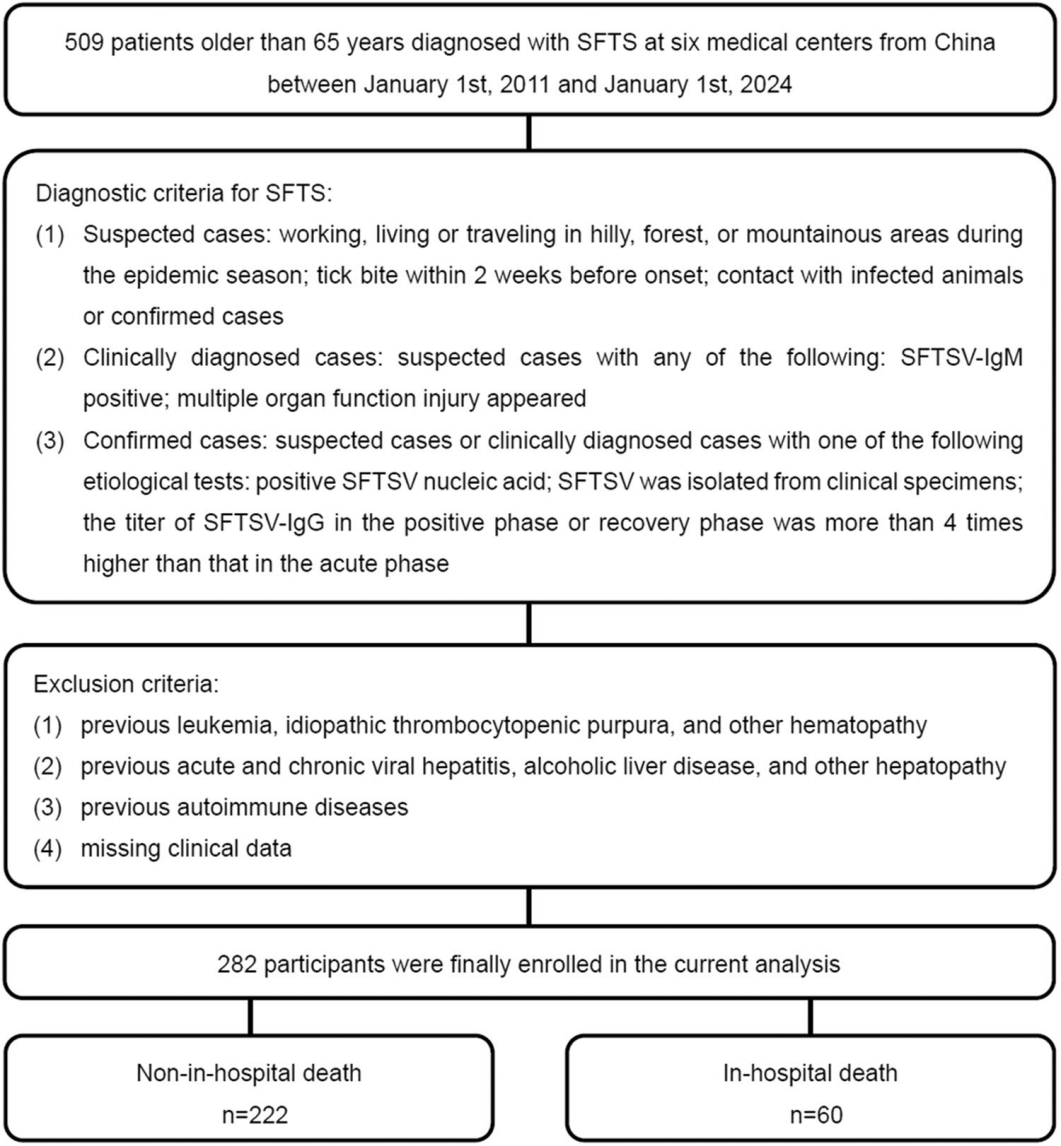
Flow diagram for the enrollment of study population. SFTS severe fever with thrombocytopenia syndrome, SFTSV SFTS virus.

We retrospectively screened patients older than 65 years diagnosed with SFTS at six infectious disease centers of China (Beijing Ditan Hospital, Yantai Qishan Hospital, Qingdao No. 6 People's Hospital, Tai'an City Central Hospital, Dalian No. 6 People's Hospital, and Dandong Infectious Disease Hospital) between January 1, 2011 and January 1, 2024. The diagnostic criteria for SFTS were summarized as follows: (1) Suspected cases: epidemiological histories: working, living or traveling in hilly, forest, or mountainous areas during the epidemic season; tick bite within 2 weeks before onset; contact with infected animals or confirmed cases. (2) Clinically diagnosed cases: suspected cases with any of the following: SFTSV‐IgM positive; multiple organ function injury appeared. (3) Confirmed cases: suspected cases or clinically diagnosed cases with one of the following etiological tests: positive SFTSV nucleic acid; SFTSV was isolated from clinical specimens; the titer of SFTSV‐IgG in the positive phase or recovery phase was more than four times higher than that in the acute phase [20]. Exclusion criteria were listed in detail in Figure 1.

The study protocol was approved by the Clinical Research Ethics Committee of Beijing Ditan Hospital, Capital Medical University. All subjects were orally informed and endorsed to participate in the present study.

### 2.2 Data Collection and Definitions

Demographic information and clinical characteristics were obtained from a self‐reported questionnaire and then filled in the database by trained staff who were ignorant of the study protocol. The first test of laboratory examination after admission, including white blood cell count (WBC), neutrophil count (NEU), neutrophil percentage (NEU%), lymphocyte count (LYM), lymphocyte percentage (LYM%), monocyte count (MONO), monocyte percentage (MONO%), hemoglobin (HGB), platelet count (PLT), prothrombin time (PT), activated partial thromboplastin time (APTT), alanine aminotransferase (ALT), aspartate aminotransferase (AST), ALB, total bilirubin (TBIL), direct bilirubin (DBIL), creatinine, blood urea nitrogen (BUN), uric acid (UA), fasting blood glucose (FBG), creatine kinase (CK), lactic dehydrogenase (LDH), and C‐reactive protein (CRP), were detected by standardized method in core laboratory. BMI was calculated as weight (kg)/[height (m)]^2^. The calculation of GNRI was referred to the former study: 14.89 × serum albumin (g/dL) + 41.7 × actual/ideal BMI (kg/m^2^) [12]. The ideal BMI was defined as a value of 22 kg/m^2^.

The observational endpoint of the current study was defined as the in‐hospital death. Time to death was defined as the time from the onset of symptoms to in‐hospital death happened.

### 2.3 Statistical Analysis

Continuous variates with normal or skewed distribution were showed as mean ± standard deviation or median (interquartile range), comparisons of which were examined by *T* test or Mann–Whitney *U* test accordingly. Nominal parameters were showed as numbers (percentages) and compared by *χ* ^2^ test or Fisher's exact test, respectively.

The cumulative survival rates of groups with lower and higher median of GNRI were described by Kaplan–Meier curve and examined by Log‐rank test. The differences between groups were examined by log‐rank test. Univariate Cox regression analyses were performed to identify potential risk factors of in‐hospital death. Four multivariate models, variates of which were chosen according to the results of univariate analysis and clinical practice, were built to explore the robustness of GNRI in predicting in‐hospital death. Variates with underlying collinearity were not introduced into models simultaneously. The hazard ratio (HR) and 95% confidence interval (CI) were estimated by regarding GNRI as a continuous or categorical variate separately.

The predictive value of GNRI for in‐hospital death was then estimated by receiver operating characteristic (ROC) analysis, whereby the area under the ROC curves (AUCs) were calculated. Moreover, the AUC and the Harrell's C‐index for respective models were evaluated and compared by *Z* test to estimate the incremental effects of GNRI on risk prediction over the established model. Furthermore, continuous net reclassification improvement (NRI) and integrated discrimination improvement (IDI) were also calculated to evaluate the incremental impact of GNRI on risk reclassification and discrimination.

Statistical analyses were performed by SPSS 26.0 (IBM, Armonk, NY, USA) and R Programming Language 3.6.3. Statistical significance was defined as two‐sided *p*‐value < 0.05.

## 3 Results

Ultimately, 282 patients with SFTS who met the study protocol were enrolled in this study, with a mean age of 68.65 ± 4.06 years, consisting of 134 (47.5%) females and 148 (52.5%) males. In total, 60 patients suffered in‐hospital death during hospitalization, with a mortality rate of 21.3%.

### 3.1 Baseline Information of the Study Population

The study population was divided into two groups according to the presence or absence of in‐hospital death. As displayed in Table 1, patients who experienced in‐hospital death were older, had lower BMI, and had longer time from onset to admission. In terms of clinical manifestations, lethargy was more common in patients who suffered in‐hospital death, whereas other clinical features did not differ significantly between the two groups. As for laboratory parameters, patients who died in hospital exhibited higher levels of NEU, NEU%, APTT, BUN, and CRP, while lower levels of LYM%, PLT, and ALB. The discrepancies in WBC, LYM, MONO, MONO%, HGB, PT, ALT, AST, TBIL, DBIL, creatine, UA, FBG, CK, and LDH were not significant.

**Table 1:** Baseline demographic and clinical characteristics of the study population.

	Total population *n* = 282	Non‐in‐hospital death *n* = 222	In‐hospital death *n* = 60	*p*
Age, years	68.65 ± 4.06	68.07 ± 3.70	70.80 ± 4.59	< 0.001
Gender, female (%)	134 (47.5)	102 (45.9)	32 (53.3)	0.309
BMI, kg/m^2^	23.07 ± 3.01	23.56 ± 2.86	21.23 ± 2.86	< 0.001
History of bites (%)	70 (24.8)	55 (24.8)	15 (25.0)	0.971
Medical history (%)				
Hypertension	19 (6.7)	16 (7.2)	3 (5.0)	0.753
Diabetes mellitus	10 (3.5)	7 (3.2)	3 (5.0)	0.770
Cardiovascular disease	7 (2.5)	6 (2.7)	1 (1.7)	> 0.999
Cerebrovascular disease	7 (2.5)	5 (2.3)	2 (3.3)	0.992
Time from onset to admission, days	6.44 ± 2.58	6.27 ± 2.41	7.05 ± 3.07	0.037
General manifestations (%)				
Pyrexia	229 (81.2)	180 (81.1)	49 (81.7)	0.918
Shiver	122 (43.3)	99 (44.6)	23 (38.3)	0.385
Dizzy	74 (26.2)	59 (26.6)	15 (25.0)	0.805
Headache	98 (34.8)	79 (35.6)	19 (31.7)	0.572
Fatigue	176 (62.4)	133 (59.9)	43 (71.7)	0.095
Myalgia	103 (36.5)	83 (37.4)	20 (33.3)	0.563
Arthralgia	76 (27.0)	61 (27.5)	15 (25.0)	0.701
Lymphadenectasis	49 (17.4)	36 (16.2)	13 (21.7)	0.323
Petechiae	23 (8.2)	16 (7.2)	7 (11.7)	0.263
Respiratory manifestations (%)				
Cough	53 (18.8)	40 (18.0)	13 (21.7)	0.521
Expectoration	46 (16.3)	35 (15.8)	11 (18.3)	0.633
Dyspnea	38 (13.5)	31 (14.0)	7 (11.7)	0.644
Gastrointestinal manifestations (%)				
Anorexia	207 (73.4)	158 (71.2)	49 (81.7)	0.103
Nausea	160 (56.7)	128 (57.7)	32 (53.3)	0.549
Emesis	77 (27.3)	62 (27.9)	15 (25.0)	0.652
Abdominal distension	26 (9.2)	21 (9.5)	5 (8.3)	0.789
Abdominal pain	54 (19.1)	41 (18.5)	13 (21.7)	0.576
Diarrhea	59 (20.9)	44 (19.8)	15 (25.0)	0.381
Melena	9 (3.2)	8 (3.6)	1 (1.7)	0.731
Neurological manifestations (%)				
Coma	2 (0.7)	1 (0.5)	1 (1.7)	0.897
Lethargy	12 (4.3)	6 (2.7)	6 (10.0)	0.013
Confusion	15 (5.3)	10 (4.5)	5 (8.3)	0.241
Dysphoria	3 (1.1)	1 (0.5)	2 (3.3)	0.222
Convulsion	14 (5.0)	9 (4.1)	5 (8.3)	0.176
Laboratory examinations				
WBC, ×10^9^/L	2.17 (1.43, 4.32)	2.13 (1.40, 3.85)	2.50 (1.60, 5.27)	0.128
NEU, ×10^9^/L	1.30 (0.80, 2.51)	1.24 (0.74, 2.34)	1.69 (0.98, 3.94)	0.029
NEU%, %	60.65 (47.63, 74.00)	57.55 (46.08, 72.33)	68.02 (54.25, 77.78)	0.008
LYM, ×10^9^/L	0.63 (0.39, 1.26)	0.66 (0.40, 1.25)	0.57 (0.32, 1.43)	0.458
LYM%, %	30.40 (19.18, 42.34)	32.37 (20.28, 43.51)	25.00 (13.76, 32.92)	0.001
MONO, ×10^9^/L	0.13 (0.07, 0.36)	0.13 (0.07, 0.34)	0.14 (0.06, 0.52)	0.867
MONO%, %	6.50 (3.93, 10.46)	6.57 (4.29, 10.54)	6.27 (3.31, 9.34)	0.219
HGB, g/L	137.5 ± 19.3	138.22 ± 19.07	134.72 ± 19.86	0.213
PLT, ×10^9^/L	52.00 (36.85, 71.00)	54.50 (39.00, 72.25)	41.70 (29.10, 65.25)	0.004
PT, s	12.83 ± 1.44	12.85 ± 1.45	12.76 ± 1.38	0.660
APTT, s	44.00 ± 12.76	43.19 ± 12.28	47.00 ± 14.10	0.040
ALT, U/L	81.05 (40.75, 141.90)	82.40 (39.75, 141.90)	74.50 (45.25, 142.75)	0.634
AST, U/L	152.00 (68.00, 300.48)	148.40 (69.10, 295.30)	154.50 (68.00, 375.50)	0.778
ALB, g/L	33.7 ± 4.9	34.49 ± 4.59	30.89 ± 4.81	< 0.001
TBIL, mg/dL	9.40 (6.70, 12.79)	9.34 (6.85, 12.79)	9.75 (5.99, 12.82)	0.533
DBIL, mg/dL	3.80 (2.60, 5.43)	3.80 (2.69, 5.30)	3.93 (2.53, 5.58)	0.797
Creatine, μmol/L	72.00 (58.00, 96.84)	70.95 (59.00, 93.25)	76.45 (56.25, 117.68)	0.227
BUN, mmol/L	5.20 (3.84, 7.15)	4.95 (3.75, 6.59)	6.51 (4.03, 9.48)	0.007
UA, μmol/L	244.00 (186.00, 298.00)	247.75 (185.00, 300.35)	232.34 (189.73, 276.74)	0.549
FBG, mmol/L	7.4 ± 2.9	7.22 ± 2.66	7.99 ± 3.45	0.115
CK, U/L	412.83 (151.50, 1093.25)	376.00 (151.50, 1014.25)	611.50 (152.00, 1599.25)	0.176
LDH, U/L	660.00 (378.00, 1269.73)	624.50 (362.25, 1215.00)	776.00 (450.75, 1544.50)	0.125
CRP, mg/L	5.55 (2.79, 14.39)	4.70 (2.61, 13.52)	8.46 (3.30, 21.23)	0.021

*Note:* Continuous variables are presented as mean ± SD and median (IQR). Nominal variables are presented as *n* (%). *p* values comparing between the group of non‐in‐hospital death and the group of in‐hospital death.

Abbreviations: ALB, albumin; ALT, alanine aminotransferase; APTT, activated partial thromboplastin time; AST, aspartate aminotransferase; BMI, body mass index; BUN, blood urea nitrogen; CK, creatinine kinase; CRP, C‐reactive protein; DBIL, direct bilirubin; FBG, fasting blood glucose; HGB, hemoglobin; LDH, lactate dehydrogenase; MONO, monocyte; MONO%, monocyte percentage; NEU, neutrophil, NEU%, neutrophil percentage; LYM, lymphocyte; LYM%, lymphocyte percentage; PLT, platelet; PT, prothrombin time; TBIL, total bilirubin; UA, uric acid; WBC, white blood cell.

**Figure 2:**
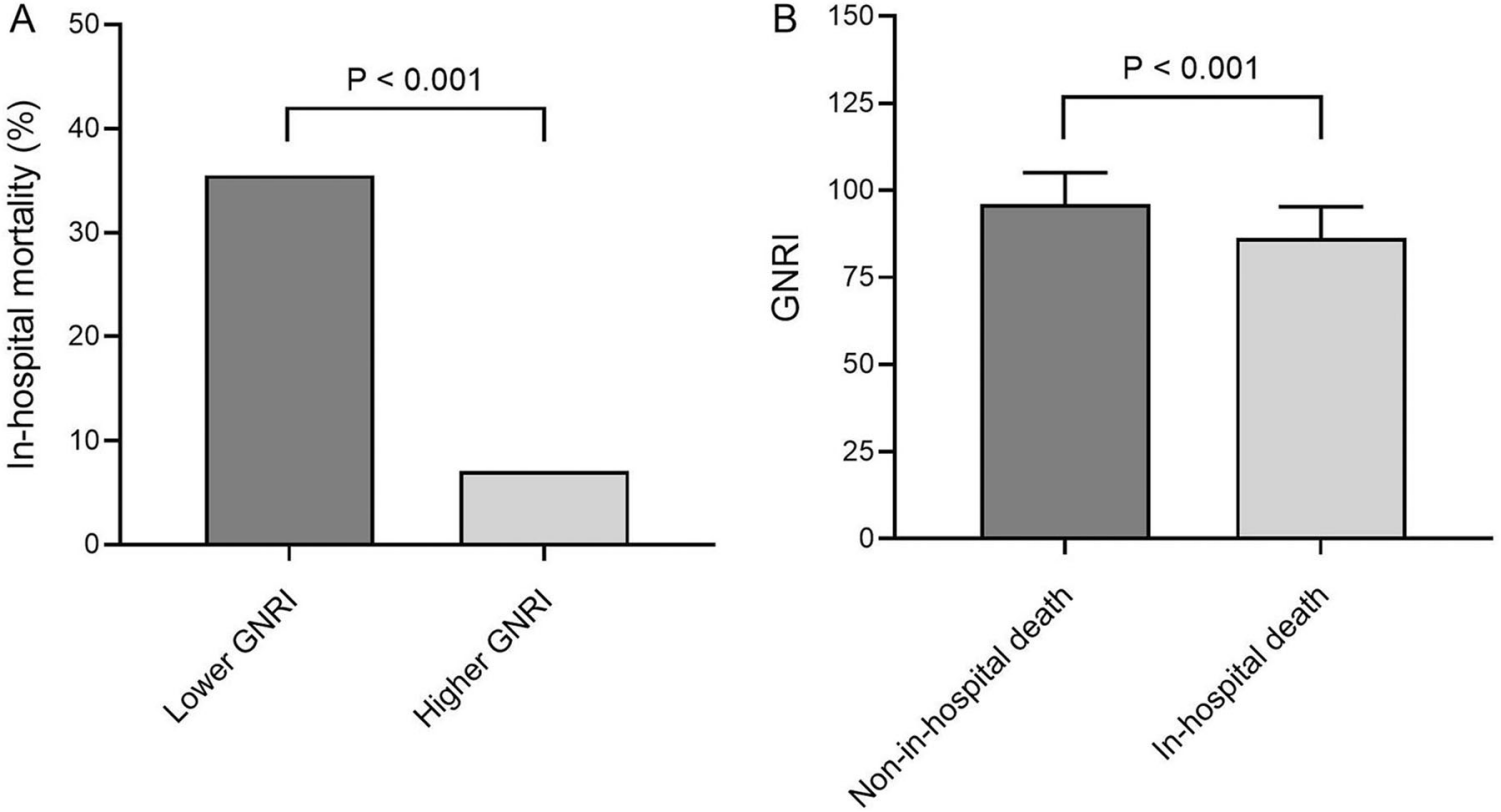
Association between GNRI and in‐hospital death. (A) the in‐hospital mortality in lower GNRI group [50 (35.5%)] is higher than higher GNRI group [10 (7.1%)] (*p* < 0.001). (B) the GNRI levels in in‐hospital death group (86.24 ± 9.03) is lower than non‐in‐hospital death group (96.02 ± 9.05) (*p* < 0.001). GNRI geriatric nutritional risk index.

Patients with lower median of GNRI showed higher in‐hospital mortality than those with higher median [50 (35.5%) vs. 10 (7.1%), *p* < 0.001] (Figure 2A). On the other hand, patients who experienced in‐hospital death during hospitalization displayed significantly lower GNRI levels than those who did not (86.24 ± 9.03 vs. 96.02 ± 9.05, *p* < 0.001) (Figure 2B).

### 3.2 Predictive Value of GNRI for In‐Hospital Death

**Figure 3:**
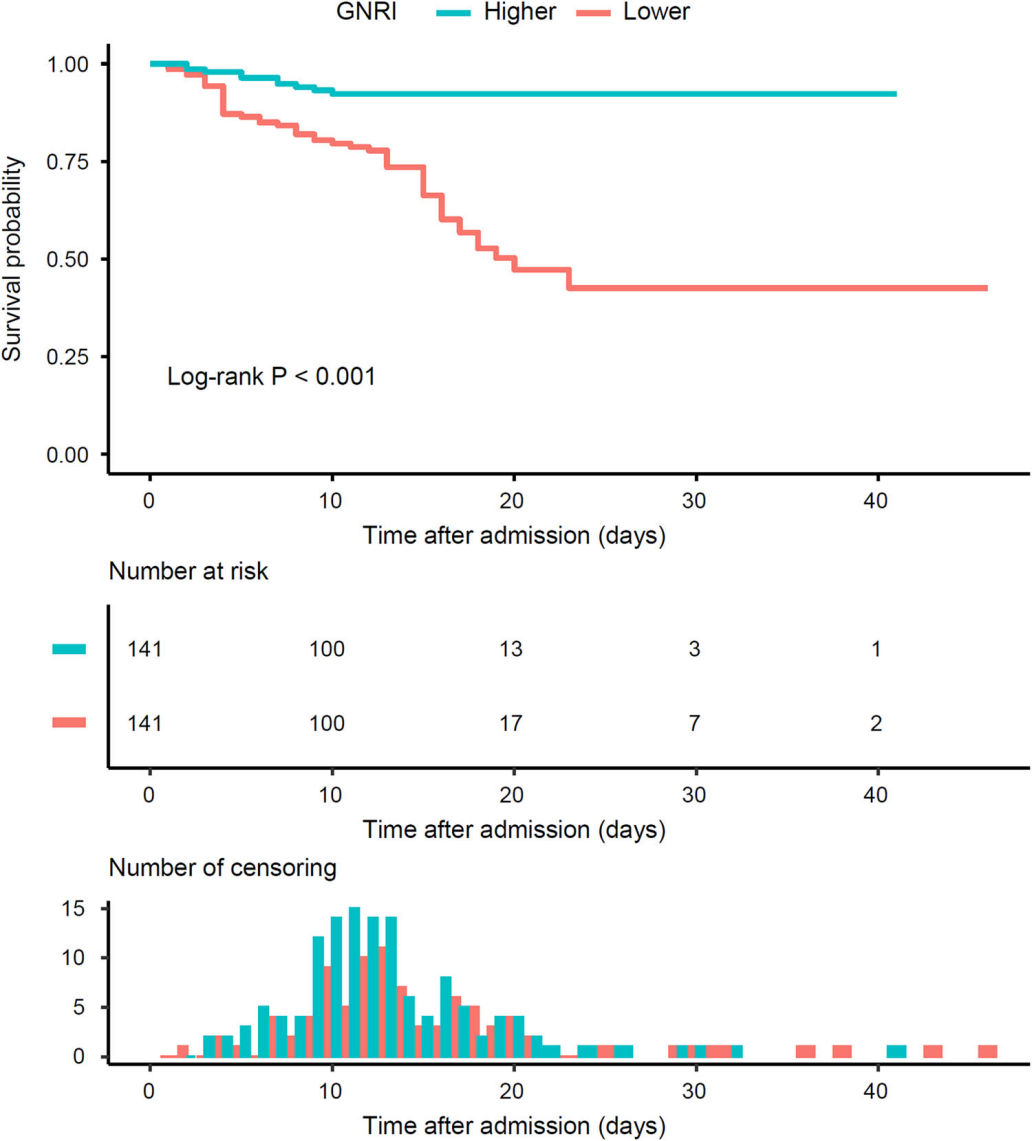
Kaplan–Meier survival curves according to the median of GNRI. The survival probability in lower median GNRI group is lower than higher median GNRI group (Log‐rank *p* < 0.001). GNRI geriatric nutritional risk index.

Kaplan–Meier analysis revealed that the cumulative survival probability of patients with lower GNRI was significantly lower than that of those with higher GNRI (Log‐rank *p* < 0.001) (Figure 3). Univariate Cox regression analysis also verified the predictive value of GNRI for in‐hospital death, in spite of taking GNRI as continuous (HR 1.083, 95% CI 1.058–1.109, *p* < 0.001) or categorical variate (HR 4.635, 95% CI 2.347–9.151, *p* < 0.001).

In univariate Cox regression analysis, variates including age, BMI, time from onset to admission, neurological manifestations, NEU%, LYM%, PLT, APTT, AST, ALB, CK, LDH, and CRP were confirmed to be potential predictors of in‐hospital death (Table S1). After taking the results of univariate analysis, clinical experiences, and previous authoritative research into consideration, four multivariate models were constructed to determine whether GNRI was an independent predictor of in‐hospital death. Despite adjustment for multiple factors, GNRI remained an independent predictor of in‐hospital death, either examining HR by evaluating 1‐unit decrease of GNRI (Model 1: HR 1.072, 95% CI 1.046–1.099, *p* < 0.001; Model 2: HR 1.072, 95% CI 1.046–1.099, *p* < 0.001; Model 3: HR 1.073, 95% CI 1.043–1.104, *p* < 0.001; Model 4: HR 1.081, 95% CI 1.044–1.121, *p* < 0.001) or by taking the higher median of GNRI as reference (Model 1: HR 4.229, 95% CI 2.137–8.368, *p* < 0.001; Model 2: HR 4.352, 95% CI 2.155–8.789, *p* < 0.001; Model 3: HR 4.043, 95% CI 1.997–8.189, *p* < 0.001; Model 4: HR 3.058, 95% CI 1.428–6.548, *p* = 0.004) (Table 2).

**Table 2:** Uni‐ and multi‐variate Cox regression analyses investigating the predictive value of GNRI for in‐hospital death.

	GNRI as continuous variate^a^			GNRI as categorical variate^b^		
	HR	95% CI	*p*	HR	95% CI	*p*
Crude model	1.083	1.058–1.109	< 0.001	4.635	2.347–9.151	< 0.001
Model 1	1.072	1.046–1.099	< 0.001	4.229	2.137–8.368	< 0.001
Model 2	1.072	1.046–1.099	< 0.001	4.352	2.155–8.789	< 0.001
Model 3	1.073	1.043–1.104	< 0.001	4.043	1.997–8.189	< 0.001
Model 4	1.081	1.044–1.121	< 0.001	3.058	1.428–6.548	0.004

*Note:* Model 1: adjusted for age, gender. Model 2: adjusted for Model 1 and hypertension, diabetes mellitus, cardiovascular disease, cerebrovascular disease. Model 3: adjusted for Model 2 and time from onset to admission, neurological manifestations. Model 4: adjusted for Model 3 and NEU%, LYM%, PLT, APTT, AST, BUN, CK, LDH, CRP.

Abbreviations: CI, confidence interval; HR, hazard ratio; GNRI, geriatric nutritional risk index.

^a^ HR was evaluated by examining 1‐unit decrease of GNRI.

^b^ HR was evaluated by taking the higher median of GNRI as reference.

### 3.3 Diagnostic Performance of GNRI for In‐Hospital Death

**Figure 4:**
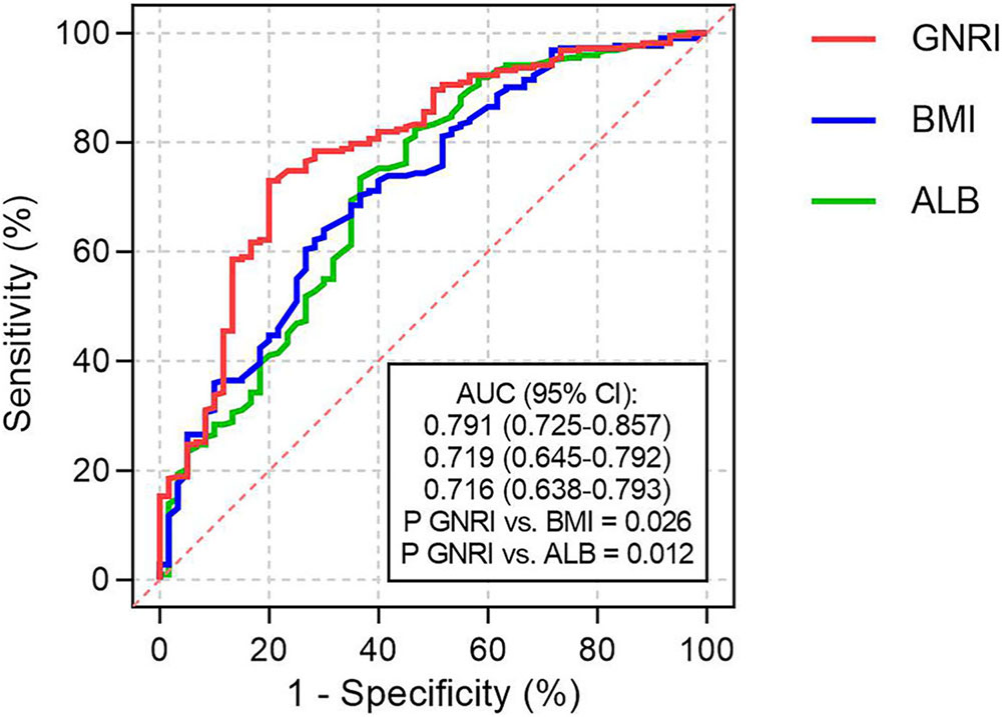
Predictive value of GNRI and its components for in‐hospital death. The AUC (95% CI) of GNRI [0.791 (0.725–0.857)] is higher than BMI [0.719 (0.645–0.792), *p* = 0.026] and ALB [0.716 (0.638–0.793), *p* = 0.012]. ALB, albumin; AUC, area under the ROC curve; BMI, body mass index; CI, confidence interval; GNRI, geriatric nutritional risk index.

The diagnostic performances of GNRI and its components for in‐hospital death were estimated by ROC analysis. The results showed that GNRI and its components all exhibited moderate‐to‐high diagnostic strength for in‐hospital death. Compared with BMI (0.719, 95% CI 0.645–0.792) and ALB (0.716, 95% CI 0.638–0.793), however, GNRI (0.791, 95% CI 0.725–0.857) displayed the highest diagnostic value for in‐hospital death, manifested as the maximum AUC (*p* for GNRI vs. BMI = 0.026; *p* for GNRI vs. ALB = 0.012) (Figure 4).

### 3.4 Incremental Effect of GNRI on the Prediction of In‐Hospital Death

**Figure 5:**
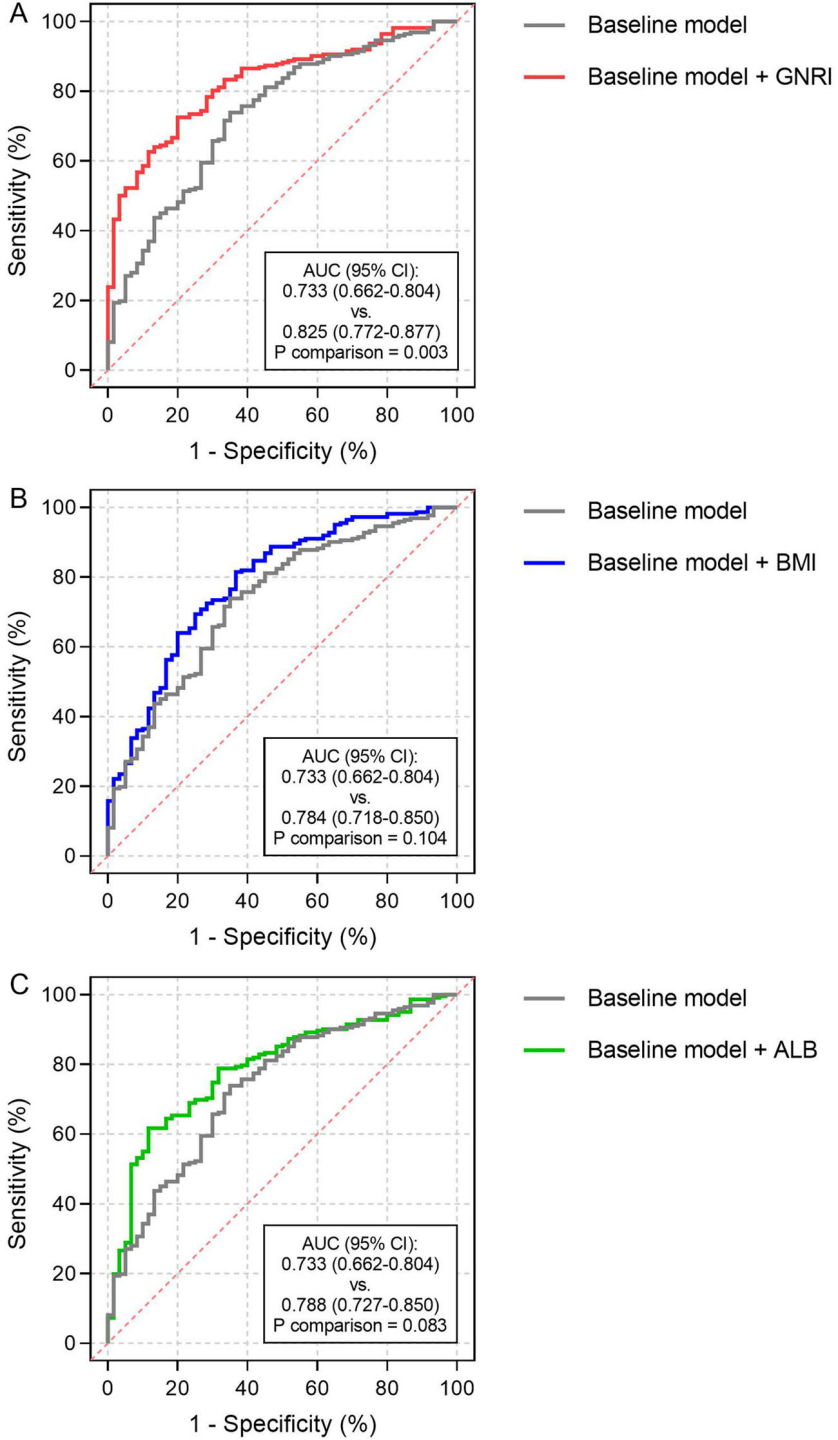
Incremental effect of GNRI and its components on the prediction of in‐hospital death. (A) introducing GNRI into the baseline model brings significant incremental effect on the prediction of in‐hospital death (*p* = 0.003). (B) introducing BMI into the baseline model has no significant incremental effect on the prediction of in‐hospital death (*p* = 0.104). (C) introducing ALB into the baseline model has no significant incremental effect on the prediction of in‐hospital death (*p* = 0.083). ALB, albumin; AUC, area under the ROC curve; BMI, body mass index; CI, confidence interval; GNRI, geriatric nutritional risk index.

Risk factors, including age, neurological manifestations, NEU%, AST, BUN, and LDH, that were identified by a previous authoritative study [21], were considered as the baseline model. After introducing GNRI into the baseline model, a significant incremental effect on the predictive performance for in‐hospital death can be seen, as indicated by an increase in AUC (baseline model: 0.733, 95% CI 0.662–0.804 vs. baseline model + GNRI 0.825, 95% CI 0.772–0.877, *p* for comparison = 0.003) (Figure 5A). However, the introduction of BMI (baseline model: 0.733, 95% CI 0.662–0.804 vs. baseline model + BMI 0.784, 95% CI 0.718–0.850, *p* for comparison = 0.104) and ALB (baseline model: 0.733, 95% CI 0.662–0.804 vs. baseline model + ALB 0.788, 95% CI 0.727–0.850, *p* for comparison = 0.083) did not bring about a significant increase in AUC (Figure 5B,C).

In addition, the introduction of GNRI into the baseline model also contributed to an increased Harrell's C‐index (baseline model: 0.753, 95% CI 0.684–0.822 vs. baseline model + GNRI 0.838, 95% CI 0.785–0.890, *p* for comparison = 0.007), significant continuous NRI (0.338, 95% CI 0.043–0.551, *p* = 0.027), and significant IDI (0.118, 95% CI 0.030–0.233, *p* = 0.007) (Table 3). The incremental effect was insignificant or relatively weak when BMI and ALB were introduced into the baseline model (details shown in Table 3).

**Table 3:** Incremental effect of GNRI and its components on risk stratification for in‐hospital death.

	Harrell's C‐index			Continuous NRI			IDI		
	Est.	95% CI	*p*	Est.	95% CI	*p*	Est.	95% CI	*p*
Baseline model^a^	0.753	0.684–0.822	—	—	—	—	—	—	—
+ GNRI	0.838	0.785–0.890	0.007	0.338	0.043–0.551	0.027	0.118	0.030–0.233	0.007
+ BMI	0.799	0.735–0.863	0.119	0.301	0.001–0.458	0.040	0.089	0.015–0.193	< 0.001
+ ALB	0.796	0.736–0.857	0.089	0.036	−0.118–0.380	0.452	0.036	−0.011–0.124	0.166

Abbreviations: ALB, albumin; BMI, body mass index; CI, confidence interval; GNRI, geriatric nutritional risk index; IDI, integrated discrimination improvement; NRI, net reclassification improvement.

^a^ The baseline model consists of age, neurological manifestations, NEU%, AST, BUN, and LDH.

## 4 Discussion

The present study initially investigated the predictive value of GNRI for in‐hospital mortality in patients with SFTS. The main research results show as follows: (1) After adjustment of multiple models, GNRI remained a significant predictor of in‐hospital death, either being taken as a nominal or continuous variate; (2) GNRI showed a moderate‐to‐high power in predicting in‐hospital death; (3) The addition of GNRI to a former established model exhibited significant improvement on the predictive value for in‐hospital death.

SFTS is a highly fatal acute infectious disease primarily found in mountainous and hilly regions. It can occur throughout the year, with a higher incidence in the spring and summer seasons. Since the first reported case in 2009, the number of cases has rapidly increased, and in 2017, the World Health Organization (WHO) listed SFTS as a priority disease for attention [22]. The main sources of infection are infected animals, although patients can also serve as sources of infection. Transmission is primarily through tick bites but can also occur through contact with the blood, secretions, or excretions of infected animals or patients. SFTSV infection disrupts immune function and can trigger a cytokine storm in severe cases, leading to death from hemorrhage or multiorgan failure. Early studies indicated a mortality rate of up to 30% [1], and the latest guidelines in China (2023) suggest a mortality rate as high as 20% [20]. In elderly patients with SFTS, the disease tends to be more severe with higher mortality rates [23]. Given the lack of vaccines and specific antiviral treatments, early identification of high‐risk individuals needing intensive care upon admission is crucial. This study is the first to analyze the relationship between nutritional status and poor prognosis in elderly patients with SFTS, showing that the GNRI is correlated with the risk of in‐hospital death in this population. Our findings indicate that elderly SFTS patients who died in the hospital had significantly lower GNRI levels compared to survivors, and the GNRI is an independent risk factor for in‐hospital death in elderly SFTS patients.

GNRI, proposed by Bouillanne et al. in 2005, is a nutrition‐related indicator used to predict the prognosis of elderly patients and is considered a predictor of in‐hospital mortality and prolonged hospital stays. GNRI is simply calculated by using serum albumin and BMI. It has been proved by previous studies to be closely associated with adverse prognosis in patients suffering from various diseases. GNRI can predict poor outcomes in elderly cancer patients, including those with head and neck cancer, pancreatic cancer, colorectal cancer, and non‐small cell lung cancer [24, 25, 26, 27]. It also has significant predictive value in elderly patients with conditions like hemodialysis or heart failure [13, 14]. As for infectious diseases, GNRI has also been demonstrated to be closely associated with adverse prognosis by former studies. Research from Li et al., which included 2834 elderly sepsis patients admitted to the ICU, found that the GNRI level at admission was significantly negatively correlated with the 28‐day mortality rate in elderly patients with septic shock [18]. Study from Wei et al. explored 346 elderly patients with severe community‐acquired pneumonia and found that the GNRI scores in the poor prognosis group were significantly lower than those in the good prognosis group, suggesting the potential of GNRI as an independent risk factor for poor prognosis in this selected population [28]. Additionally, the relationship between GNRI and poor outcomes in elderly patients with pyogenic liver abscesses and the occurrence of invasive candidiasis in ICU elderly patients has also been revealed by previous studies [19, 29], indicating that GNRI may be a significant risk factor for predicting the incidence of adverse outcomes in elderly pyogenic liver abscess patients and invasive candidiasis in elderly ICU patients. As mentioned above, GNRI has been shown to be associated with prognosis in various infectious diseases, however, no research targeting at the impact of GNRI on prognosis in elderly patients with SFTS have been conducted currently. The present study, which explored the predictive value of GNRI for in‐hospital mortality in elderly SFTS patients for the first time, fills the gap of previous research in this field. More importantly, the results also revealed that introducing GNRI into existing mortality prediction models proposed by former study [21] significantly enhanced the predictive power for in‐hospital death, indicating GNRI may provide additional information for risk prediction and stratification in elder SFTS patients.

Malnutrition is very common among the elderly and has been recognized as a challenging health issue, significantly correlated with increased mortality and morbidity. Previous studies have shown that malnutrition adversely affects the clinical outcomes of diseases, trauma, and surgery and is associated with increased incidence and mortality of various acute and chronic diseases [30, 31]. Malnutrition predisposes individuals to infectious diseases, which can worsen malnutrition, creating a vicious cycle and increasing short‐term mortality risk from diseases. Serum albumin and BMI can both indicate nutritional status; however, ALB and BMI can be influenced by non‐nutritional factors such as inflammation and renal function. Therefore, the GNRI, as a combined indicator of serum albumin and BMI, addresses these limitations and enhances diagnostic accuracy. The GNRI has the largest area under the ROC curve compared to BMI and serum albumin, making it statistically significant.

In infectious diseases, targeting the pathogen is crucial, but for many acute viral diseases like dengue fever and hemorrhagic fever with renal syndrome, there are still no specific antiviral drugs. Similarly, for SFTS, there are no vaccines or effective antiviral treatments available, making symptomatic supportive treatment particularly important. Our research results provide a basis for further exploration of nutritional interventions to improve the prognosis of elderly patients with SFTS. Considering the correlation between GNRI and in‐hospital mortality in elderly SFTS patients, nutritional therapy could be considered for high‐risk patients. By providing high‐nutritional‐value food or nutritional solutions, we can supplement nutrition and maintain good nutritional status in high‐risk patients. Future researchers could consider conducting prospective interventional trials to validate whether nutritional interventions can improve the prognosis of elderly patients with SFTS.

This study has some limitations. Firstly, this study is a retrospective observational study, with a relatively small sample size, and the results of this study have not been validated with a new cohort, which may significantly limit its statistical power. Further prospective cohort studies with relatively larger scale of population, which may provide stronger statistical power and more reliable results, are needed to be conducted in our future work. Secondly, continuous monitoring of albumin and BMI during hospitalization may provide a more accurate and comprehensive reflection of patients' nutritional status and disease progression. In our study, only single albumin and BMI levels were included at admission, which may lead to a non‐real‐time assessment of patients' nutritional status and disease progression, thereby affecting the interpretation of the findings. In future studies, taking dynamic monitoring of albumin and BMI levels during hospitalization into account is necessary for improving the reliability of the study results. Thirdly, measurements of viral load and/or antibody titers, which can provide more valuable information, are not available in this database. Future prospective studies that include viral load and antibody titers will be investigated to explore more comprehensive results. Finally, the study's endpoint is defined as in‐hospital mortality, and post‐discharge follow‐up for adverse events is not included, so it is unclear whether GNRI is a predictor of long‐term adverse prognosis. Further studies including long‐term follow‐up data are required to provide a more complete picture of patient outcomes and then explore whether GNRI continues to be a predictor beyond the hospital setting.

## 5 Conclusion

GNRI is closely related to the risk of in‐hospital death in patients with SFTS. GNRI, which is calculated from albumin and BMI, may provide additional information in identifying patients at high risk of suffering adverse prognosis, thus can be regarded as a significant risk predictor in clinical practice.

## Author Contributions

Tingyu Zhang and Zhihai Chen made substantial contributions to study design, data analysis, and manuscript writing. Yanli Xu made substantial contributions to intellectual direction and manuscript revision. Ziruo Ge, Di Tian, Chenxi Zhao, Ling Lin, and Zhensheng Liu made substantial contributions to data collection and follow‐up. Qi Zhao made substantial contributions to data analysis. All authors have read and approved the final manuscript.

## Ethics Statement

The study protocol was endorsed by the Clinical Research Ethics Committee of Beijing Ditan Hospital, Capital Medical University. All subjects were informed and agreed to participate in the present study.

## Consent

The authors have nothing to report.

## Conflicts of Interest

The authors declare no conflicts of interest.

## Supporting information

Supporting information.

## Data Availability

The data that support the findings of this study are available from the corresponding author upon reasonable request.
