# Supplementary material for: Geriatric Nutritional Risk Index Plays Important Role in Predicting In‐Hospital Mortality in Patients With Severe Fever With Thrombocytopenia Syndrome: A Multi‐Center Observational Study
Source: J Med Virol. 2025 Feb 18;97(2):e70252. doi: 10.1002/jmv.70252 (PMC11834140; doi:10.1002/jmv.70252)
Supplement: Supplementary file 1 — Supporting information. [file JMV-97-e70252-s001.docx]

## Table S1. Univariate COX proportional hazard regression evaluating potential predictors of in-hospital death

|  | HR | 95% CI | P |
| --- | --- | --- | --- |
| Age | 1.104 | 1.053-1.158 | <0.001 |
| Gender | 1.187 | 0.715-1.973 | 0.507 |
| BMI | 0.794 | 0.725-0.870 | <0.001 |
| History of bites | 1.032 | 0.575-1.852 | 0.915 |
| Hypertension | 0.792 | 0.248-2.530 | 0.693 |
| Diabetes mellitus | 1.835 | 0.571-5.900 | 0.308 |
| Cardiovascular disease | 0.653 | 0.090-4.719 | 0.673 |
| Cerebrovascular Disease | 1.254 | 0.306-5.147 | 0.753 |
| Time from onset to admission | 1.119 | 1.019-1.228 | 0.019 |
| Neurological manifestations | 2.158 | 1.214-3.836 | 0.009 |
| WBC | 1.021 | 0.971-1.073 | 0.416 |
| NEU% | 1.018 | 1.001-1.035 | 0.034 |
| LYM% | 0.974 | 0.954-0.994 | 0.010 |
| MONO% | 1.008 | 0.964-1.054 | 0.723 |
| HGB | 0.995 | 0.982-1.007 | 0.406 |
| PLT | 0.989 | 0.979-0.999 | 0.034 |
| PT | 0.927 | 0.779-1.104 | 0.395 |
| APTT | 1.016 | 1.001-1.031 | 0.042 |
| ALT | 1.001 | 0.999-1.002 | 0.225 |
| AST | 1.001 | 1.000-1.001 | 0.001 |
| ALB | 0.875 | 0.828-0.926 | <0.001 |
| TBIL | 1.007 | 0.981-1.034 | 0.596 |
| DBIL | 1.020 | 0.989-1.053 | 0.209 |
| Creatine | 1.001 | 0.999-1.003 | 0.288 |
| BUN | 1.032 | 0.998-1.067 | 0.068 |
| UA | 1.000 | 0.998-1.002 | 0.820 |
| FBG | 0.999 | 0.987-1.011 | 0.815 |
| CK | 1.000 | 1.000-1.001 | 0.007 |
| LDH | 1.000 | 1.000-1.001 | 0.037 |
| CRP | 1.017 | 1.000-1.034 | 0.048 |

BMI body mass index, WBC white blood cell, NEU% neutrophil percentage, LYM% lymphocyte percentage, MONO% monocyte percentage, HGB hemoglobin, PLT platelet, PT prothrombin time, APTT activated partial thromboplastin time, ALT alanine aminotransferase, AST aspartate aminotransferase, ALB albumin, TBIL total bilirubin, DBIL direct bilirubin, BUN blood urea nitrogen, UA uric acid, FBG fasting blood glucose, CK creatinine kinase, LDH lactate dehydrogenase, CRP C-reactive protein, HR hazard ratio, CI confidence interval
